# Targeting Macrophages as a Potential Therapeutic Intervention: Impact on Inflammatory Diseases and Cancer

**DOI:** 10.3390/ijms19071953

**Published:** 2018-07-04

**Authors:** Mirco Ponzoni, Fabio Pastorino, Daniela Di Paolo, Patrizia Perri, Chiara Brignole

**Affiliations:** Laboratory of Experimental Therapy in Oncology, Istituto Giannina Gaslini, Via G. Gaslini 5, 16147 Genoa, Italy; fabiopastorino@gaslini.org (F.P.); danieladipaolo@gaslini.org (D.D.P.); patriziaperri@gaslini.org (P.P.)

**Keywords:** macrophage targeting, macrophage depletion, nanomedicine, preclinical models, inflammatory diseases, cancer

## Abstract

Macrophages, cells belonging to the innate immune system, present a high plasticity grade, being able to change their phenotype in response to environmental stimuli. They play central roles during development, homeostatic tissue processes, tissue repair, and immunity. Furthermore, it is recognized that macrophages are involved in chronic inflammation and that they play central roles in inflammatory diseases and cancer. Due to their large involvement in the pathogenesis of several types of human diseases, macrophages are considered to be relevant therapeutic targets. Nanotechnology-based systems have attracted a lot of attention in this field, gaining a pivotal role as useful moieties to target macrophages in diseased tissues. Among the different approaches that can target macrophages, the most radical is represented by their depletion, commonly obtained by means of clodronate-containing liposomal formulations and/or depleting antibodies. These strategies have produced encouraging results in experimental mouse models. In this review, we focus on macrophage targeting, based on the results so far obtained in preclinical models of inflammatory diseases and cancer. Pros and cons of these therapeutic interventions will be highlighted.

## 1. Introduction

Macrophages, an essential component of the innate immune system, present heterogeneous functional phenotypes correlated to a high plasticity grade [1]. They play central roles in both physiological and pathological conditions, being involved in developmental and homeostatic tissue processes and in a plethora of human diseases. As a consequence, they have been deeply studied, and the idea to exploit them as therapeutic targets has focused the interest of experts of different research areas from inflammation to cancer.

At present, it is widely accepted that a variety of diseases (i.e., diabetes, atherosclerosis, rheumatoid arthritis, obesity, and cancer) are associated with chronic inflammation [2]. Furthermore, it is recognized that macrophages are the major players of chronic inflammation in most human diseases [1,3]. The knowledge about their biology, mechanisms of action, and activation phenotypes has been largely increased in the last few years. Macrophages have an innate and elevated propensity to adapt to the neighboring microenvironment and to rapidly change in response to environmental stimuli. Thus, it appears difficult to find out and design a unique therapeutic strategy based on macrophage modulation that is easily applicable to different kinds of human pathologies.

In the last decade, many studies have been addressed to understanding the mechanisms involved in macrophage activation and to relate them to macrophage function. The well-known bipolar model [4,5] distinguishes macrophages into two main different subsets: pro-inflammatory (M1, classically activated via interferon-γ) and anti-inflammatory (M2, alternatively activated via interleukin 4 and 13). However, a vast literature suggests that this is only a simplification, with M1 and M2 macrophages representing only the two extremes of a huge spectrum of activation states [6,7]. Indeed, this classification takes into consideration neither the fact that M1 and M2 stimuli do not exist as single entities in tissues, nor the source and the context of environmental stimuli. Moreover, the dichotomy of M1 and M2 macrophages derives from the pre-genomic era, when few markers were considered to establish differences and similarities in macrophage responses to stimuli [6]. Finally, it is important to underline that macrophages can also develop mixed M1/M2 phenotypes in different pathological conditions, where it is possible to find macrophages with intermediate activation status [8,9].

Due to their large involvement in several high-impact diseases, macrophages have acquired a lot of interest as potential therapeutic targets. With this focus, several approaches have been developed, ranging from depletion to re-programming/re-polarization [10]. In this field, nanotechnology-based systems (i.e., liposomes, dendrimers, gold nanoparticles, and polymeric nanoparticles) have attracted attention [11,12], also due to their potential to be modified according to the disease site to be targeted. Furthermore, they have been demonstrated to be more effective than conventional delivery systems and with limited side effects [13]. In line with this, the understanding of the biology and the activation states of macrophages in different disease conditions has been, and will be in the near future, very helpful to design therapeutic protocols of personalized medicine.

Here, we reexamine the data obtained in preclinical mouse models of inflammatory diseases and cancer. In particular, the targeting of macrophages led to encouraging results, highlighting the possibility of translating these therapeutic interventions to the clinical practice.

## 2. Macrophages in Health and Disease

Macrophages are innate immune cells characterized by evolutionarily conserved functions, such as host defense against infection and tissue homeostasis. They were first identified by Metchnikoff in the late 19th century as tissue-residing immune cells belonging to the reticulum endothelial system and endowed with the ability to detect and phagocytose dying cells, harmful molecules, and pathogens [14,15]. A new classification was made in the 1970s, when van Furth and colleagues proposed macrophages as being part of the mononuclear phagocytic system (MPS) [16].

Based on their differential localization within the body, macrophages were also classified in discrete subsets as tissue-resident macrophages (TRM) [17]. They present different transcriptional profiles, associated to heterogeneous phenotypes, as a consequence of the tissue-specific function [7]. Primarily described as immune sentinels able to protect the tissue in which they are located, TRM are characterized by several steady-state functions. Indeed, in healthy tissues, they are responsible for homeostasis maintenance through the clearance of senescent cells and regulation of tissue metabolism. Based on their host tissue, they were defined as Langerhans cells in the skin, Kupffer cells in the liver, microglia within the brain, alveolar macrophages in the lung, peritoneal macrophages in the peritoneum, and osteoclasts in the bone. Initially, it was supposed that TRM were derived from circulating blood monocytes. At present, a new concept suggests that they can derive either from embryonic progenitors, seeding the tissues during development and being able to maintain their population independently from blood-borne precursors in adulthood, or from hematopoietic stem cell-derived circulating monocytes [18].

For many years, it has been accepted as a paradigm model that macrophages can adopt opposite activation states in response to environmental stimuli sensed primarily by Toll-like receptors. Based on this assumption, they have been distinguished as classically (M1) and alternatively (M2) activated macrophages. M1 and M2 macrophages are pro-inflammatory and anti-inflammatory, respectively. The main activities associated to M1/M2 phenotypes can be summarized as “Fight” for M1 macrophages and “Fix” for M2 macrophages. These two diverse programs take place as a response to environmental stimuli through enzymatic pathways responsible for arginine catabolism [5]. Macrophages that follow the “Fight” program, thanks to inducible nitric oxide synthase (iNOS), convert arginine to citrulline and nitric oxide (NO), inhibiting cell proliferation and inducing cancer cell death. On the other hand, macrophages activating the “Fix” program are able to promote cell proliferation and repair, via the metabolism of arginine through arginase I (ArgI), that produces ornithine and urea.

Notably, it is clear that, at present, the extraordinary plasticity of macrophages is not limited to these two opposed phenotypes, representing only the extremes of a wide and continuous array of activation states [19,20].

The concept that macrophages take part as major players in a series of human diseases associated with chronic inflammation has been a matter of debate for a long time. At present, much evidence supports this theory and a vast literature is dedicated to this field. Furthermore, many efforts have been made to understand the role of macrophages in inflammation and cancer and to reveal the different phenotypes they can acquire in inflammation-driven pathological conditions. Although the dichotomy of M1 versus M2 does not entirely satisfy the reality, it is acknowledged that under pathological conditions, macrophages can be found in both the activation states.

In the last decade, researchers have tried to decipher which polarization/phenotype is associated to a specific inflammation-related disease. The findings obtained led to the understanding that, in most of the pathological conditions analyzed, macrophages can temporally acquire different phenotypes, due to the evolution of the disease.

### 2.1. Inflammatory Diseases

Several studies evidenced the importance of macrophages in diseases such as asthma, atherosclerosis, rheumatoid arthritis, osteoarthritis, endometriosis, diabetes type 1 and 2, and obesity.

In asthma, Zaslona and colleagues demonstrated the role of alveolar macrophages, concluding that resident alveolar macrophages play a protective role during the early phase of allergic lung inflammation. On the other hand, they stated that the recruited monocytes, putative precursors of alveolar macrophages, are involved in asthma pathogenesis by promoting lung inflammation [21]. Moreover, Jiang and colleagues recently updated the role of macrophages in asthma, concluding that both M1 and M2 subsets participate in asthma pathogenesis, being alternately predominant in the different phases of the disease [22]. The aforementioned situation, with M1 and M2 macrophages that alternate their predominance under pathological conditions, is found in several other human pathologies.

In atherosclerosis, a chronic inflammatory disease characterized by the narrowing and thickening of the arteries caused by buildup of plaque around the artery wall, macrophages participate in all the stages of the disease, being involved in pathogenesis and progression [23]. Summarily, pro-inflammatory macrophages are implicated in plaque initiation and progression, while the anti-inflammatory ones are involved in plaque stabilization. Furthermore, it has been reported that both macrophage subtypes (M1/M2) can be found in atherosclerotic plaques, where they are differently distributed [24].

In rheumatoid arthritis, a common autoimmune disorder characterized by chronic arthritis that leads to joint destruction, macrophages have a leading role in pathogenesis and disease progression. It has been reported that an increased number of sublining macrophages in the synovium is an early hallmark of active rheumatic disease. Furthermore, the degree of synovial macrophage infiltration is strictly related to joint erosion [25]. In the context of rheumatoid arthritis, the phenotype of the macrophages is heterogeneous and still needs to be completely elucidated. Of note, in synovial tissues and fluids, macrophages can present both pro-inflammatory and anti-inflammatory phenotypes [26].

In osteoarthritis, not commonly recognized as an inflammatory disease, macrophages directly participate in synovial inflammation through the production of TNF-α and IL-1β and, indirectly, through the activation of synovial fibroblasts [27].

Recently, the contribution of macrophages in metabolic diseases such as diabetes [28] and in obesity-related adipose inflammation, frequently associated with the development of insulin resistance and type 2 diabetes, has been also reported [29].

### 2.2. Cancer

Inflammatory cells have been recognized as a key component of the tumor microenvironment (TME), which can be represented as an ecological niche playing a critical role in cancer development, progression, and control. Within this niche, a complex interplay between different cell types (proliferating cancer cells, stromal cells, and infiltrating immune cells) leads to the establishment of a microenvironment, whose components influence each other to promote tumor progression. Macrophages are the dominant portion of infiltrating leukocytes in all tumors, where they are defined as tumor-associated macrophages (TAMs) and are mostly characterized by an M2-like phenotype [30]. They mainly derive from circulating monocytes, recruited at the tumor site by chemotactic factors.

Macrophages participate in both tumor initiation and development/progression. It is now accepted that non-malignant TME cells, being macrophages the most prominent ones, play a tumor-initiating role, generating a persistent inflammatory response against environmental stimuli [31,32]. Indeed, it has been reported that different inflammatory conditions (i.e., inflammatory bowel disease, Crohn’s disease, asbestosis, etc.) are highly correlated with the increased risk of cancer development [33]. Noteworthy, macrophages involved in the phase of tumor initiation are characterized by a pro-inflammatory phenotype. Once the tumor is established, macrophages switch to an anti-inflammatory phenotype. It is customary to say that macrophages are educated by neoplastic cells to acquire a protumor phenotype [32,34,35].

TAMs support tumor progression through different mechanisms, promoting angiogenesis and tumor spreading at distant sites and creating an immunosuppressive environment [36]. Furthermore, it seems that different subsets of TAMs can be found at the tumor site, where they can be either temporally distinguished, depending on disease evolution, or geographically determined on the basis of their location. In this regard, macrophages found in hypoxic areas of the tumor are prevalently involved in tumor angiogenesis, while those residing at the tumor–stroma interface take part in invasion and metastasis [30,37].

TAMs are abundant in established tumors and their presence is often associated with increased tumor progression and invasion [32]. Indeed, preclinical and clinical evidence shows that a prevalence of TAMs within tumors is associated with a worse overall prognosis in a high proportion of solid tumors (i.e., breast cancer, ovarian cancer, melanoma, etc.; Table 1) [38,39,40,41,42,43,44]. Until now, to our knowledge, only in a few cases (i.e., colorectal cancer and non-small cell lung cancer) it has been reported that a high density of macrophages is associated with improved overall survival [45,46,47]. Doubtful is the role of TAMs in gastric cancer; a study by Ohno and colleagues proves, indeed, their positive correlation with improved survival [48], while more recent papers demonstrate that an high infiltration of TAMs is a predictor of poor prognosis for gastric cancer patients [49,50].

## 3. Macrophage Targeting

Despite macrophages being characterized by an elevated grade of plasticity, they have been the interest of researchers as potential therapeutic targets. They can acquire different phenotypes, frequently overlapping each others, in response to the continuous environmental stimuli they are subjected to. For these reasons, the design of therapeutic interventions has been very challenging, due most of all to the lack of unambiguous markers that can identify and distinguish pathological macrophages from their safe and useful counterparts. Several strategies have been pursued and adopted with the aim of manipulating macrophages, and their re-education and depletion can be considered the most important methods. In this scenario, nanotechnology-based systems have been greatly implicated and have led to important results in preclinical models [51,52,53].

### 3.1. Macrophage Re-Education

Macrophage re-education is one of the most promising strategies realized in the field of anticancer therapy, aimed at re-programming M2-like, protumor TAMs to M1-like macrophages endowed with antitumor functions [36,54]. In this field, nanotechnology-based systems have been explored, resulting as being applicable for successful preclinical achievements [12]. Recently, Song and colleagues [55] used bioconjugated manganese dioxide nanoparticles to efficiently prime TAMs to an antitumor M1-like phenotype, also resulting in higher sensitivity and response to chemotherapy, via the reduction of tumor hypoxia, in a murine breast cancer model.

Furthermore, Zanganeh and colleagues demonstrated that ferumoxytal nanoparticles significantly inhibited tumor growth in a breast cancer model, with an increased number of M1 macrophages at the tumor site [56]. In the same work, ferumoxital nanoparticles were able to inhibit liver and lung metastases in a mouse model of small cell lung cancer. Also in this case, metastases reduction was paralleled by the reversal of TAMs to the tumoricidal M1 phenotype.

An interesting study performed by Ortega and colleagues [57] demonstrated that mannosylated nanoparticles (Mn-NPs), encapsulating small interfering RNAs (siRNAs) against IκBα, were able to selectively target TAMs via the mannose receptor (CD206) and to restore the NF-κB signaling pathways, finally resulting in the induction of cytotoxic and immunostimulatory functions of TAMs in vitro. In a previous work, the same authors demonstrated that Mn-NPs are biocompatible and able to enhance the uptake of TAMs in vivo [58]. This last finding enables one to envisage that the proposed Mn-NPs could be translated to the in vivo setting.

Huang and colleagues developed a nucleic acid-encapsulating carrier endowed with two important features: (1) selectivity for TAMs (targeted to the macrophage galactose-type lectin I) and (2) microenvironment-responsiveness (pH-sensitive). These nanocarriers, encapsulating anti-IL-10 and anti-IL-10R oligonucleotides, were tested in a preclinical mouse model of liver cancer, and were demonstrated to be effective in suppressing the protumor functions of TAMs, stimulating their antitumor activities [59].

### 3.2. Macrophage Depletion

In pathological conditions, the more radical approach used to block macrophages is represented by their direct killing, named “macrophage depletion”. This strategy is based on the use of either depleting antibodies, such as anti-colony-stimulating factor 1 receptor (anti-CSF1R), or molecules exerting a specific toxicity against macrophages, such as bisphosphonates (i.e., clodronate and zoledronic acid) and trabectedin.

#### 3.2.1. Monoclonal Antibodies to the Colony-Stimulating Factor 1 Receptor

In a recent study performed in colorectal adenocarcinoma and fibrosarcoma mouse models, the depletion of TAMs was obtained by the use of a high-affinity humanized anti-colony-stimulating factor 1 receptor (CSF1R) monoclonal antibody (RG7155) [60]. The treatment with RG7155 induced a selective apoptosis of CSF1R^+^CD163^+^ M2-like macrophages, but not of CD80^+^ M1-like macrophages, in vitro, and depleted TAMs in tumor-bearing mice. This was also paralleled by an increase of cytotoxic effector CD8^+^ T cells, followed by delayed tumor growth and metastases formation.

In cervical and mammary carcinoma mouse models, the depletion of TAMs, obtained by means of a highly selective CSF1R inhibitor, resulted in the arrest and delay of tumor growth, respectively. Moreover, the depletion of macrophages was paralleled by the increase of CD8^+^ T cells infiltrating cervical and breast carcinomas [61].

More recently, the evaluation of the safety, pharmacokinetics, pharmacodynamics, and antitumor activity of a fully human antibody against CSF1R (AMG 820) in a first-in-humans phase-I study for advanced solid tumors was published [62]. This study demonstrated that the anti-CSF1R antibody AMG 820 was tolerated with manageable toxicity up to 20 mg/kg every two weeks. Nevertheless, the treatment with AMG 820 as the single agent resulted in limited antitumor activity.

#### 3.2.2. Liposomal Bisphosphonates

Liposomes, self-closed structures characterized by one or more phospholipid bilayers delimiting an inner aqueous space, were developed many years ago, and soon after demonstrated their great potential as vehicles for drug delivery [63,64,65]. Since their discovery more than 50 years ago, they have been a matter of extensive investigation, finding applicability in different medical areas. At present, due to both biological and technological features, they are considered to be the most successful drug delivery systems developed to date [66]. Several liposomal formulations are in clinical trials and some of them have been already released on the market for the cure of different kind of diseases [65,67].

The use of liposomes for drug delivery and their consequent therapeutic efficacy strictly depend on their physicochemical properties, with size and membrane charge being the most important ones [65]. Indeed, the fate of administered liposomes is closely associated to their physical features. For these reasons, researchers have been focusing their attention on the optimization of methods to synthesize liposomes suitable for in vivo use. Indeed, the inherent liposome composition renders them as extremely versatile and safe tools for medical purposes, due to their high biocompatibility and biodegradability, low immunogenicity, and drug protection.

Depending on the nature of the diseased tissues to be targeted, it is important to take into account the overall dimension of the liposome-based cargos [68,69]. Indeed, inflammatory or solid tumor tissues are characterized by enhanced vascular permeability. This feature can be exploited by nanocarriers of appropriate size (namely 50–100 nm), which can passively enter the leaky vessels of pathological tissues, accumulating at the target site. This phenomenon is known as the enhanced permeability and retention effect (EPR) and it is of particular interest for drug-loaded liposomes used for cancer treatment [70,71]. On the other hand, larger liposomes easily interact with plasma proteins undergoing opsonisation, a prerequisite for their recognition and capture by cells of the reticulum endothelial system (RES). The uptake of liposomes by phagocytes of the RES can be prevented by the surface modification of the liposomes themselves. Regarding this matter, the most used method is represented by the insertion of polyethylene glycol (PEG) chains at the liposome surface with the aid of cross-linker lipids [72,73,74]. Outer membrane modification with PEG (a method defined as “PEGylation”) confers to liposomes stealth features, leading to increased blood circulation time, a great characteristic for drug-delivery systems suitable for application in cancer therapy [75].

As underlined before, liposomes present the inherent property to target mononuclear phagocytic cells. This feature can be exploited in the case of a macrophage-targeted therapy. In this regard, large multilamellar clodronate-contained liposomes were developed by van Rooijen and colleagues in the 1990s as specific tools for the transient depletion of macrophages [76]. Clodronate belongs to the drug family of bisphosphonates and is used for the treatment of osteolytic bone disease and post menopausal osteoporosis because of its ability to inhibit osteoclast function [77].

Large clodronate liposomes, due to their big size, are rapidly recognized and taken up by macrophages. Once captured and ingested by them, clodronate liposomes are exposed to lysosomal phospholipases that, after degradation of the phospholipid bilayer, lead to the release of the entrapped drug within the cytoplasmic compartment of the cells. This, in turn, causes apoptotic cell death via an ATP-dependent mechanism [76]. This technique is called “liposome-mediated macrophage suicide”.

Clodronate liposomes can efficiently deplete tissue-resident macrophages of different body districts, preferentially depending on the route of administration [78]. Indeed, Kupffer cells, spleen macrophages, and bone marrow macrophages can be depleted by intravenous injection (*i.v.*) of clodronate liposomes, while intraperitoneal injection (*i.p.*) is needed for peritoneal macrophages. Moreover, the subcutaneous (*s.c.*) administration is able to deplete macrophages of the draining lymph nodes.

### 3.3. Nanomedicine and Macrophage Functionality: Disease Models

This section focuses on the application of macrophage depletion in a series of mouse disease models.

#### 3.3.1. Inflammatory Diseases

Rheumatoid arthritis is one of the first inflammatory diseases in which liposomal clodronate was applied [79,80]. In experimental mouse models of arthritis, a single intraarticular injection of clodronate liposomes determined the reversible depletion of synovial phagocytic cells, accompanied by the reduction of cartilage destruction [79,80]. In an open study conducted on rheumatoid arthritis-affected patients scheduled for knee joint replacement, a single intraarticular injection of clodronate liposomes led to effective macrophage depletion in the synovial lining [81]. Moreover, the depletion of macrophages in the lining was also paralleled by a significant reduction of the expression of adhesion molecules, such as ICAM-1 and VCAM-1. These molecules are widely expressed in the synovium of rheumatoid arthritis patients. Of note, the procedure was well tolerated and resulted as being nontoxic. This last study represents, to our knowledge, the only example of macrophage depletion performed in humans. The results obtained are very important and encouraging for further investigation in this field and for designing protocols to be applied in the treatment of rheumatoid arthritis.

In a study conducted by Bu and colleagues, it was concluded that visceral adipose tissue macrophages (VATMs) can be regarded as a potential target for the development of drugs to be used for both the prevention and therapy of obesity and obesity-related complications [82]. Indeed, in a high-fat-diet animal model, the depletion of VATMs obtained by intraperitoneal injection of clodronate liposomes blocked the high-fat-induced weight gain and the development of insulin resistance. Gene expression analysis demonstrated that VATMs depletion was associated to the downregulation of genes involved in lipogenesis and gluconeogenesis.

Similar results were also obtained by Feng and colleagues, which demonstrated that the intraperitoneal injection of clodronate liposomes in diet-induced obese (DIO) mice reduced VATMs and improved systemic glucose homeostasis and insulin sensitivity. This was also paralleled by increased blood levels of the insulin-sensitizing hormone adiponectin [83].

Endometriosis, although its pathogenesis is still matter of debate, can be considered to be a chronic inflammatory disease that affects a large fraction of menstruating women. It is characterized by the growth of vascularized endometrial tissue in aberrant locations outside of the uterus, principally the pelvis, and it is associated with symptoms such as chronic pelvic pain, dysmenorrhea, and reduced fertility [84,85]. The endometriosis process is also accompanied by altered immune surveillance in the local peritoneal microenvironment. It has been reported that infiltrating macrophages are abundant in endometriotic lesions, where they contribute to the production of elevated amounts of pro-inflammatory and chemotactic cytokines.

In this context, the depletion of macrophages by the use of clodronate liposomes was very useful to understand their role in the establishment and development of the disease. In an experimental mouse model of endometriosis [86], clodronate liposomes were *i.p.* injected at different time points, with respect to the implantation of the endometrium. Two different treatment protocols were performed. In the first one, macrophages were depleted at early times; specifically, clodronate liposomes were injected at days 0, 4, and 8 from the implantation of the endometrial tissue in recipient mice. Clodronate liposomes significantly reduced the percentage of F4/80- and CD11b-positive cells in the peritoneum of sacrificed animals, with respect to mice treated with PBS-containing liposomes. This was also accompanied by a significant reduction in the weight of endometriotic lesions. These data indicate that, in the absence of macrophages, the syngeneic endometrium retains the ability to adhere to the peritoneal layer; nevertheless, the lesions fail to grow. In the second set of experiments, macrophages were depleted at later times (4 and 8 days after lesion engraftment), when endometriotic lesions had already been established and organized. In this case, macrophages depletion did not affect the number of endometriotic lesions, which was similar in treated and untreated animals. However, the total weight of lesions was significantly lower in mice subjected to ablative treatment. The results obtained indicated that the recruitment of the macrophages into the lesions is not only an early event sufficient for the initial development of the lesions, but it is also a necessary step for their subsequent successful establishment. These findings are straightforward because they suggest that it could be possible to treat human endometriosis by depleting local macrophages. Furthermore, it has been established that macrophages in the peritoneal fluid and/or those infiltrating the endometriotic lesions of patients express markers of alternative activation, such as CD163 (hemoglobin scavenger receptor) and CD206. In the forthcoming future, it can be envisaged to develop new formulations of clodronate liposomes directly targeted to these antigens. Due to the fact that macrophages have also a protective function and are involved in homeostatic tissue processes, the possibility of selectively targeting a specific subset of them is of particular interest for the development of patient-tailored treatment protocols.

Several studies have been also conducted on inflammatory diseases of the lung, such as granulomatous inflammation in response to *Mycobacterium tuberculosis* and chronic obstructive pulmonary disease (COPD). COPD is a life-threatening inflammatory disease of the lung characterized by chronic airway inflammation, mucus hypersecretion, and airway remodeling. In a cigarette smoke-induced COPD mouse model, Beckett and colleagues provided evidence for the pivotal role of macrophages in the pathogenesis of the disease [87]. Indeed, macrophage depletion in the lung, obtained by means of intranasal administration of clodronate liposomes given along an 8-week period of smoke exposure, resulted in a reduced smoke-induced epithelial thickening and emphysema development. Furthermore, macrophage depletion also determined protection against alteration of the lung function.

In a mouse model of pulmonary tuberculosis, it was demonstrated that the depletion of alveolar macrophages (AM), achieved by intranasal delivery of clodronate liposomes, was able to protect mice from lethality. AM depletion was associated with reduced outgrowth of mycobacteria in lungs and liver and to a polarized production of type-I cytokines in the lung tissue; moreover, AM-depleted mice displayed deficient granuloma formation. These results provide new insights for the design of novel therapeutic strategies against intracellular bacterial diseases [88].

#### 3.3.2. Cancer

The direct killing of TAMs is a strategy aimed at eradicating macrophages at tumor sites, with the intention to abolish and/or interrupt the network of signals that favors tumor growth and progression.

Zhan and colleagues used a glucomannan polysaccharide with high affinity for the mannose receptor to specifically deliver alendronate (ALN) to TAMs. In both in vitro and in vivo experiments, this ALN–glucomannan bioconjugated formulation preferentially accumulated into macrophages, leading them to undergo apoptosis. Furthermore, in a subcutaneous sarcoma mouse model, the intratumor injection of ALN–glucomannan bioconjugated formulation determined the effective depletion of TAMs at the tumor site, reduced angiogenesis, led to the recovery of local immune surveillance, and significantly reduced tumor progression [89].

Hattori and colleagues tested the effectiveness of folate (FL)-decorated zoledronic acid (ZOL)-encapsulating liposomes in inhibiting tumor angiogenesis and tumor growth in a murine colon adenocarcinoma model. Here, although the FL–ZOL liposomes were able to induce selective cytotoxicity in vitro via the folate receptor, a severe toxicity limited their use in vivo [90].

Clodronate liposomes have been successfully applied in various mouse models of cancer with the aim of depleting macrophages at the tumor site. Banciu and colleagues demonstrated that the depletion of TAMs in a murine melanoma model was associated with a reduction of tumor volume [91]. Moreover, in a teratocarcinoma model, the depletion of TAMs resulted in the inhibition of tumor angiogenesis and in the inhibition of tumor growth [92]. In a chemically induced mouse lung adenocarcinoma model, the effective depletion of TAMs by means of clodronate liposomes was paralleled by a 50% reduction of tumor burden and tumor cell proliferation, as detected by the Ki67 marker, with respect to control animals treated with a vehicle only [93]. In this model, the treatment was more effective in slowing the growth of larger tumors, while the overall number of lung tumor nodules was not affected, being similar in treated and control mice.

Nevertheless, one of the major concerns of the use of clodronate liposomes to deplete TAMs relies on the fact that ablating strategies may also compromise the protective functions of macrophages themselves. Indeed, therapeutic approaches based on macrophage depletion have been associated with an increased risk of infection [94]. For this reason, our laboratory recently developed a new clodronate-containing liposomal formulation able to reach the tumor site without being massively detected and taken up by the cells of the MPS. The development of liposomes of appropriate physicochemical properties (i.e., size, Z-potential, and membrane modification) leads to the depletion of macrophages at different sites. Indeed, as already described before, large-sized liposomes are rapidly taken up by phagocytic cells [65], representing an excellent tool to preferentially target tissue macrophages rather than TAMs. Thus, to safely reach the tumor site, avoiding early recognition by the MPS, liposomes are subjected to PEGylation [74,95].

In a recent study, Piaggio and colleagues developed a new liposomal formulation of clodronate (Clo-Lipo-DOTAP) characterized by stealth features and small size, necessary to gain long circulating time and EPR-mediated tumor targeting properties, respectively. Moreover, the new formulation was not toxic (blood levels of kidney and liver markers were not altered), was cell-specific (immune cells other than macrophages were not affected), and resulted effective at clodronate dosage from 5 to 10 times lower than that used in previous studies [53].

Clo-Lipo-DOTAP were used in two xenograft mouse models of melanoma, the first one resembling the hallmarks of early stage primary tumor, and the second representing the lung metastatic behavior of advanced melanoma [53]. In both cases, the treatment with Clo-Lipo-DOTAP showed inhibition of tumor growth. In the primary tumor model, the depletion of TAMs determined a reduction of the volume of the subcutaneous tumors; this was further accompanied by a reduced tumor vascularization. In the metastatic melanoma model, the antitumor effect driven by TAMs depletion was evident in the significant decrease of pulmonary tumor nodules, also paralleled by a reduction of tumor microvessel density.

Plasma levels of a number of cytokines, chemokines, and growth factors involved in and/or related to TAMs polarization, chemoattraction, and functions (IL-10, Mo KC, TNF-α, VEGF, and PDGF-bb) were statistically decreased in Clo-Lipo-DOTAP-treated mice with respect to controls. This result further highlights the antitumor efficacy of this new formulation.

Interestingly, tumor masses of treated animals, although significantly smaller with respect to controls, present a proliferating index comparable to that derived from tumors of untreated mice [53], as underlined by a similar percentage of Ki67-positive nuclei. These findings clearly indicate that the antitumor effectiveness, obtained after the depletion of TAMs, was derived from the inhibition of tumor angiogenesis and from the reduction of inflammatory-related signals, while it was not the result of direct tumor cell killing. All together, these data are encouraging and suggest that this approach could be used, in combination with first-line therapy, for the treatment of advanced-stage melanoma. Indeed, despite the recent application of promising protocols based on immunotherapy and targeted therapy [96], melanoma continues to be a very challenging tumor (Table 2).

## 4. Conclusions and Future Perspectives

The nanotechnology-based systems described in this review and intended to manipulate macrophages in both inflammatory diseases and cancer have been demonstrated to be beneficial in improving the outcome in preclinical mouse models, holding promise for the future design of therapeutic interventions (Table 3). However, it appears evident that macrophage-targeted therapies are intrinsically characterized by limitations. Indeed, their effectiveness is closely related to an in-progress therapeutic window. In fact, the phenotypic re-reversal and re-population of macrophages in the case of re-education and depletion strategies, respectively, can occur after the discontinuance of the treatment.

Moreover, the overall challenge that nanotechnology-based systems have to face is represented by their poor selectivity. The continuous changes in phenotype/activation state that macrophages adopt in response to the constant environmental stimuli result in the unspecific targeting of tissue-resident macrophages, which play a pivotal role in tissue homeostasis and body defense. Indeed, the depletion of macrophages is frequently associated to increased risk of infection [97].

The aforementioned limitations determine the need to find novel treatments able to balance the cost–benefit ratio of this approach. More durable and specifically targeted interventions might be necessary to obtain long-lasting results, while sparing resident macrophages. In light of new insights on the activation states and related markers of macrophages in several human diseases, it is reasonable to hypothesize that it will be feasible to develop, in the near future, new nanocarriers targeted to specific subsets of macrophages involved in the pathogenesis and/or progression of a given pathology.

As a note of caution, we think that further investigations are needed to attempt translating the results so far obtained to the clinic. Indeed, despite the positive results obtained in preclinical studies and the proof-of-concept clinical study performed on rheumatoid arthritis [81], to our knowledge, no approved clinical trials are currently running and/or recruiting patients. The lack of translation to the clinical practice could be due to the intrinsic limitation related to the method of macrophage depletion and to the main obstacles usually faced by nanodelivery systems (i.e., pharmaceutical manufacturing, government regulations, intellectual properties), issues already reviewed elsewhere [75]. Moreover, the phase-I study performed with the use of the anti-CSF1R antibody also revealed its limits, supporting the hypothesis that this therapeutic approach might be applied only in a combination therapy [62]. In our opinion, a treatment strategy based on the combination of macrophage re-programming and macrophage depletion, which could result in a more deep and long-lasting microenvironment priming, should lead to improved therapeutic results in both inflammatory diseases and cancer. In this regard, a nanotechnology-based system could be an exceptionally suitable moiety. The development of nanocarriers decorated with macrophage-specific ligands and loaded with a combination of molecules for both macrophage killing and re-programming would, indeed, represents a true step ahead to design innovative protocols for macrophage-driven pathologies. This would pave the way for future patient-tailored therapies.

## Figures and Tables

**Table 1:** Prognostic significance of macrophages in tumors. TAMs: tumor-associated macrophages, M1:pro-inflammatory, M2: anti-inflammatory.

Cancer Type	Prognosis Association	References
Melanoma	M2 TAMs accumulation in malignant melanoma was confirmed as biomarker of poor prognosis.	[43] Falleni, M. et al.
Breast	High density of TAMs was associated with malignant phenotype and poor survival in breast cancer patients, emerging as a novel prognostic factor.	[40] Zhao, X. et al.
Ovarian Cancer	High CD163^+^ TAMs infiltration was associated with poor prognosis, while a high M1/M2 ratio predicted better prognosis.	[41] Yuan, X. et al.
Bladder Cancer	A high TAMs count was associated with a lower 5-year survival rate with respect to low TAMs count, and it was proposed as a predictor of clinical outcome.	[44] Hanada, T. et al.
Colorectal Cancer	Macrophage infiltration was associated with improved patient survival. No difference in prognosis was found in patients with different ratios of M1/M2 infiltration.	[45] Edin, S. et al.
Non-small cell lung cancer	Independently of the density of CD68^+^ TAMs, the localization and M1/M2 polarization were suggested as potential prognostic markers.	[42] Mei, J. et al.

**Table 2:** Nanomedicine and macrophage functionality: macrophage depletion in disease models. *i.t.*: intratracheal; *i.v.*: intravenous; *s.c.*: subcutaneous; *i.p.*: intraperitoneum; *i.a.*: intraarticular.

Disease Model	Clodronate Liposomal (Administration Schedule)	Achievements	References
Rheumatoid arthritis	(1) Single *i.a.* injection of clodronate liposomes in a mouse model of arthritis. (2) Single *i.a.* injection of clodronate liposomes in rheumatoid arthritis-affected patients	(1) The reversible depletion of synovial macrophages was accompanied by the reduction of cartilage destruction. (2) The effective depletion of macrophages in the synovial lining was paralleled by the reduction of adhesion molecules (ICAM-1 and VCAM-1 in the lining).	[79] van Lent P.L. et al. [80] van Lent P.L. et al. [81] Barrera P. et al.
Endometriosis	I treatment schedule: clodronate liposomes were *i.p.* injected at days 0, 4, and 8 with respect to the injection of endometrial tissues in recipient mice. II treatment schedule: clodronate liposomes were *i.p.* injected at days 4 and 8 with respect to endometriotic lesions implantation.	I schedule: significant reduction of F4/80^+^ and CD11b^+^ cells in the peritoneum of sacrificed mice was observed with respect to those treated with PBS-containing liposomes. Reduction in the weight of endometriotic lesions. II schedule: the delayed treatment did not affect the number of endometriotic lesions, while their total weight was statistically reduced.	[86] Bacci M. et al.
Lung adenocarcinoma (chemically-induced mouse lung adenocarcinoma)	Single *i.t.* instillation followed by *i.v.* injection, starting 2 days after *i.t.* administration and continuing once weekly for 5 consecutive weeks.	After 4–6 weeks of clodronate liposome treatment, a 50% decrease in the number of alveolar macrophages was observed. Tumour burden was reduced by 50% compared to vehicle-treated mice. The proliferation index of tumour cells (Ki67) was also attenuated.	[93] Fritz J.M. et al.
Melanoma (B16/F10 mouse melanoma model)	Clo-Lipo-DOTAP formulation was administered *i.v.*, starting 7 days after B16/F10 cells inoculum, and was repeated 3 times with 3-day interval between injections. The same schedule was used for both *s.c.* and metastatic model.	*s.c.* model: depletion of TAMs was associated with a reduction of tumor volumes and reduced tumor vascularization. Metastatic model: reduction of pulmonary tumor nodules and decreased tumor microvessel density was observed.	[53] Piaggio F. et al.

**Table 3:** Nanotechnology-based systems and macrophage manipulation in preclinical models. ODNs: oligonucleotides.

Type of Nanocarriers		Pre-Clinical Models	Results	References
**Passive targeting**	Ferumoxytal nanoparticles	Breast cancer Small cell lung cancer	Inhibition of tumor growth (breast cancer) and liver and lung metastases (small cell lung cancer) mediated by reversal of TAMs to tumoricidal M1 macrophages.	[56] Zanganeh, S. et al.
	Clodronate Liposomes	Rheumatoid arthritis	Depletion of macrophages obtained by intra-articular injection of clodronate liposomes led to the reduction of cartilage destruction.	[79] van Lent, P.L. et al. [80] Van Lent, P.L. et al.
	Clodronate Liposomes	Obesity	Depletion of VATMs obtained by intraperitoneal injection of clodronate liposomes resulted in the block of high-fat-induced weight gain and insulin resistance	[82] Bu, L. et al.
	Clodronate Liposomes	Endometriosis	Depletion of macrophages obtained by intraperitoneal injection of clodronate liposomes resulted in weight reduction of endometriotic lesions	[86] Bacci, M. et al.
	Clodronate Liposomes	Melanoma	Depletion of TAMs was associated with a reduction of tumor volume	[89] Banciu M. et al.
	Clodronate Liposomes	Teratocarcinoma	Depletion of TAMs was associated with the inhibition of tumor angiogenesis and tumor growth	[92] Zeisberger, S.M. et al.
	Clo-Lipo-DOTAP	Melanoma	Depletion of TAMs was associated with a reduction of primary tumor growth and number of lung tumor nodules paralleled by the inhibition of tumor angiogenesis.	[53] Piaggio, F. et al.
**Active targeting**	Mannosylated nanoparticles encapsulating siRNAs against IκBα	Ex vivo macrophages	The restoration of NF-κB signaling pathways determined the induction of cytotoxic and immunostimulatory activities of TAMs	[57] Ortega, R.A. et al.
	Galactose-type lectin I-decorated nanocarriers encapsulating IL-10 and IL-10R ODNs	Liver cancer	Suppression of protumor functions of TAMs led to induction of their antitumor activities with final inhibition of tumor growth.	[59] Huang, Z. et al.
	ALN–glucomannan bioconjugates	Sarcoma	TAM depletion led to the local recovery of the immune surveillance at the tumor site and resulted in reduced tumor progression.	[89] Zhan, X. et al.
	Folate-decorated zoledronic acid-containing liposomes	Colon adenocarcinoma	Zoledronic liposomes targeted to the folate receptor were able to induce selective cytotoxicity in vitro; nevertheless, their use in vivo was limited due to the severe toxicity of zoledronic acid.	[90] Hattori, Y. et al.
